# Transfusion-transmitted infections

**DOI:** 10.1186/1479-5876-5-25

**Published:** 2007-06-06

**Authors:** Florian Bihl, Damiano Castelli, Francesco Marincola, Roger Y Dodd, Christian Brander

**Affiliations:** 1 Partners AIDS Research Center, Massachusetts General Hospital, Harvard Medical School, Boston, MA, USA; 2 Swiss Red Cross Blood Transfusion Service of Southern Switzerland, Lugano, Switzerland; 3 NIH Clinical Center, HLA Typing Laboratory, Bethesda, MD, USA; 4 American Red Cross, Holland Laboratory, Rockville, MD, USA

## Abstract

Although the risk of transfusion-transmitted infections today is lower than ever, the supply of safe blood products remains subject to contamination with known and yet to be identified human pathogens. Only continuous improvement and implementation of donor selection, sensitive screening tests and effective inactivation procedures can ensure the elimination, or at least reduction, of the risk of acquiring transfusion transmitted infections. In addition, ongoing education and up-to-date information regarding infectious agents that are potentially transmitted via blood components is necessary to promote the reporting of adverse events, an important component of transfusion transmitted disease surveillance. Thus, the collaboration of all parties involved in transfusion medicine, including national haemovigilance systems, is crucial for protecting a secure blood product supply from known and emerging blood-borne pathogens.

## Background

Although there are early reports in the history of medicine that describe attempts to treat patients with human or animal blood products, transfusion medicine is a relatively young field that has developed only since the second half of the last century. Very rapidly, however, it became clear that these therapeutic approaches also carried their problems, such as the (in-)compatibility of red blood cells and plasma between donors and recipients, and the possibility of transmitting infectious diseases [1,2]. While in the past, the risk of transfusion-transmitted infections (TTI) was accepted by patients and physicians as unavoidable, a low-risk blood supply is expected today. Since the early nineteen sixties, blood banks, as well as plasma manufacturing industries, have aggressively pursued strategies to reduce the risks of TTI. In particular, donor exclusion criteria, such as a history of hepatitis or transfusions in the past six months have been in place since early on. Today, donor evaluation, laboratory screening tests and pathogen inactivation procedures are considered crucial tools to reduce the risk of TTI, but do not completely eliminate all risk. At the same time these advances have moved transfusion medicine towards increasingly safer products, at steadily escalating costs and thus leading to major differences in transfusion product safety between wealthy and poor countries.

**Figure 1:**
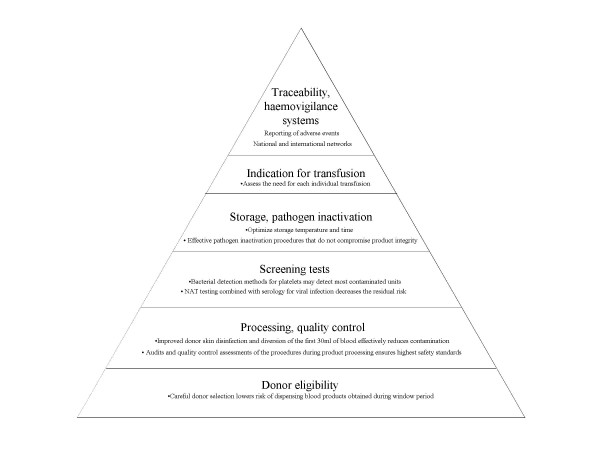
Strategies to reduce risk of transfusion transmitted infections.

The current efforts and strategies have greatly helped reduce transfusion-associated risks. Indeed, the risk of being infected by a contaminated blood unit today is orders of magnitude lower when compared to thirty years ago (Table 1). A considerable portion of this improvement is due to the introduction of nucleic acid testing (NAT), rather than relying solely on measuring pathogen-specific humoral immune responses in the donor [3]. In order to maintain the integrity, purity and adequacy of the blood supply new donor screening assays, donor deferral and pathogen inactivation of blood components need to be balanced against the undue loss of potential donors because of overly stringent exclusion criteria. These efforts would ideally be supported by national and international haemovigilance networks that help identify emerging new TTI threats; by facilitating quality assurance, quality control and the ability to monitor all steps in the transfusion chain (Figure 1) [4-6].

**Table 1:** Relative Risk of the most frequent TTI

Risk factor/infectious agent		Risk of *TTI *in blood products released		Ref.
		U.S.	Europe	
**Virus**				
HIV		1 in 2,135,000	1 in 909,000 – 5,500,000	26, 30, 33
HCV		1 in 1,930,000	1 in 2,00,000 – 4,400,000	26, 30, 33
HBV		1 in 277,000	1 in 72,000 – 1,100,000*	33
WNV		1 in 350,000	No reported cases	56, 63
HTLV-II		1 in 2,993,000	Not tested	30
**Bacteria**				
Bacterial contamination	RBC	1 in 38,500		8, 10
	Platelets	1 in 5,000		13
**Parasites**				
Malaria		1 in 1,000,000 – 5,000,000		105

* High variations between low-endemic areas and intermediate-endemic areas

## Bacterial infections

The risk of bacterial infection has emerged as the major cause of transfusion related morbidity and mortality, in part due to the reduction of other risks [4,7-10]. Bacterial contamination is more frequent in platelet concentrates (PLT) than in red blood components most likely because many microorganisms can survive and propagate under the storage conditions typically used for PLT (20–24°C), but less so for RBC (1–6°C) [8-11]

As a consequence of the increasing awareness and clinical relevance of bacterial contamination of blood components, the AABB (formerly The American Association of Blood Banks) released standards to diminish bacterial TTI [12] in 2004. In particular, bacterial testing of platelets was suggested as an effective measure to improve transfusion safety. Three testing systems are licensed in the U.S. and are in use in many transfusion centers around the world. These screening tests appear quite effective and recent data indicate that the frequency of bacterial contamination has declined by about 50% or more with contamination being detected in about one in 5,000 apheresis PLT concentrates tested[13]. The estimated incidence rates of bacterial TTI with clinical consequences range from one in 70,000 to 118,000 transfused PLT, largely depending on the amount of bacteria transfused and the type of bacterium and its pathogenicity [13,8,14].

In order to design effective strategies to reduce bacterial TTI, the bacterial infections are frequently divided based on the origin of the microorganisms: differentiating infections that originate from the environment, from the skin of the transfused subject, or from those that are likely derived from a donor bacteremia. Most commonly, contamination occurs during blood collection (insufficient disinfection of venipuncture site), or during handling of blood products (leaky seals) [15]. As a result, the most predominant bacteria isolated are usually commensals of the skin or gastrointestinal tract flora. A report from the American Red Cross on detection of bacterial contamination in platelets showed that the majority of isolates were Gram-positive aerobic pathogens (nearly 75%), in line with the organisms identified in platelet units implicated in cases of transfusion-associated sepsis (56% Gram-positive aerobes) [7].

Measures to reduce the risk of bacterial contamination focus on different steps in the transfusion chain (Figure 1) and can be classified into six aspects:

1.) Donor eligibility: To reduce asymptomatic donor bacteremia, subjects with recent dental treatments, minor surgery or increased body temperature at presentation should be excluded from donation.

2.) Optimal product processing, handling and storage: Continuous training and supervision of the responsible personnel for donation and product processing are key elements for high quality standards and product safety. Also, consistent storage temperatures (4°C for RBC and 22–24°C for PLT) need to be maintained to ensure product integrity.

3.) Skin preparation: Improved donor arm disinfection has been shown to be crucial in reducing the numbers of remaining bacteria on the phlebotomy puncture site [16-18].

4.) Removal of the initial whole blood collection (diversion): It has been shown that removal of the first 30–40 ml of whole blood from the collection bag might reduce the contamination risk from skin bacteria. In fact, improved donor arm disinfection in association with blood diversion has been reported to reduce the risk of bacterial contamination by up to 77% [19-21].

5.) Bacterial detection methods: Different methods have been investigated for detecting bacteria in platelet products prior to transfusion, including an automated bacterial culture method (BacT/ALERT system, bioMérieux), direct bacterial staining, bacterial endotoxin and ribosomal assays, nucleic acids testing for bacterial DNA, and measures of O_2 _consumption or CO_2 _production (Pall BDS, Pall Corporation) [22-25]. However, none of these detection methods seems to identify all bacterial contaminations and additional bacterial screening tests as well as better timing of bacterial testing (i.e. closer to the time of transfusion) might be needed to further improve the likelihood of correctly identifying bacterially contaminated blood products.

6.) Pathogen reduction methods: Pathogen reduction is a pro-active approach to further reduce the risk of TTI and could prove effective for most known and emerging pathogens. The goal of pathogen inactivation is to reduce transmissible pathogens (bacteria, viruses and protozoa) without compromising therapeutic efficacy of the blood product or introducing secondary risks. These techniques and their current limitations are discussed in more detail below.

## Transfusion-transmitted viral infections

Over the two last decades, much attention has been given to the prevention of transfusion-transmitted viral infections such as HIV-1 and -2, human T cell lymphotropic virus (HTLV) I and II, hepatitis C virus (HCV), hepatitis B virus (HBV) and West Nile Virus (WNV). Given the potential transmission of viruses during the 'immunological window period' [i.e. the period of early infectivity when an immunologic test is non-reactive], novel non-serology based approaches such as viral nucleic acid testing (NAT) have been established. Today NAT is performed on minipools of plasma from 16–24 donations and has significantly increased the sensitivity to detect infected blood components as it reveals viral agents earlier in the 'window period' than antibody or antigen assays[26]. However, it has some limitations in blood components with very low levels of viremia, which can even escape detection by NAT [27]. Despite this limitation, the combination of both serological testing and NAT has considerably reduced the risk of viral transmission by blood transfusion [28-30].

### HIV and HCV

Surveillance studies in Europe and in the U.S. have documented a significant reduction in the risk of HCV and HIV transmission through blood products over the last three decades [30-32]. In the mid 1980's, anti-HIV serological testing was introduced, followed a few years later by similar approaches for HCV. In 1995, the European plasma fractionating industry introduced viral NAT as a method to further ensure the integrity of virus interdiction and between 1998 and 2001, the new screening methods were widely introduced in many additional countries[31,33]. The implementation of viral NAT testing has greatly helped to reduce the residual risk of viral transmission during the 'window period' by reducing the time for effective detection from 22 days (with solely serological testing) to 11 days for HIV and from 70 to 10 days for HCV [30,34]. As a consequence, the estimated risk for HIV transmission to date is between 0.14 – 1.1 and for HCV between 0.10 – 2.33 per million units transfused[30,35-40].

As shown in a recent study assessing the risks of transfusion-mediated HIV and HCV infections between 1999 and 2003[26], the benefits of NAT testing over antibody testing were confirmed by showing that one out of 230,000 donations tested positive for HCV RNA but not for anti-HCV antibodies. For HIV, with an apparently shorter window period, one out of 3.1 million donations was RNA positive but antibody negative. However, the observed NAT detection rates and its relative benefits can vary widely between sites. For instance, in Europe only 54 anti-HCV antibody negative, HCV RNA positive samples were identified among 58 million donations tested (NAT yield: 0.93 NAT reactive sample per million antibody negative donations) compared to North America (NAT yield: 3.92/million donations) or the Pacific area (NAT yield: 2.37/million donations)[33]. In addition, NAT detection rates may be affected by viral sequence polymorphisms and next generation NAT tests need to be designed to effectively cope with increasing global viral diversity, especially for highly variable pathogens such as HIV and HCV[41].

### HBV

The risk of TT HBV infection has been continuously reduced since the introduction of the hepatitis B surface antigen (HbsAg) testing in the early 1970's, but with more than 300 million individuals infected world wide, HBV remains a considerable risk for TT infection. HBV surface antigen (HbsAg), the main screening target, is routinely included in the donor screening, but fails to detect the presence of HBV during the 'window period'. A number of countries have also added the testing for antibodies directed against the HBV core protein (anti-Hbc) to the standard screening in an attempt to detect chronic virus carriers with low-level viremia who may not have detectable HBsAg levels. Today, the residual risk of TT HBV infection varies between 0.75 per million blood donations in Australia, 3.6 – 8.5 in the USA and Canada, 0.91 – 8.7 in Northern Europe, 7.5 – 13.9 in Southern Europe up to 200 per million donations in Hong Kong, largely reflecting the global epidemiology of HBV[33]. Even though at present, no regulations for mandatory testing of blood components for HBV NAT exist, a number of countries with low HBV prevalence have implemented HBV NAT testing in plasma pools[33,35,42,43]. However, there is no evidence, so far, that pooled testing with HBV NAT is superior to the most sensitive tests for HBsAg.

The kinetics of viral antigen and antibody appearance during HBV infection create two different window periods in which one or the other test may fail: the "early acute phase", when serological markers are still negative and the "late chronic phase" when HBsAg may become gradually undetectable, although infectivity remains [44,45]. Thus, the effective immunological window period could be longer than what is generally considered (median of 59 days)[45-47]. NAT could potentially identify some of these cases and may also be of particular benefit in the detection of HBV DNA in "occult HBV infection", where HBV DNA is present in the plasma in the absence of detectable HBsAg and variable presence of anti-HBc and/or anti-HBs antibodies [48,49]. In addition, in some cases, infection by HBV mutants that affect the surface antigen conformation may result in failure to detect HBV infection by routine diagnostic assays [50-52]. Even though HBV NAT could detect these cases, higher levels of sensitivity for NAT may be necessary to cope with the characteristically low level viremia (< 500 IU/ml) seen in occult HBV infection [53-56].

### West Nile Virus

West Nile virus (WNV), a mosquito-borne RNA virus of the flavivirus family, is an emerging TT agent with potential future importance for the North American continent. WNV was first isolated in samples obtained in 1937 from patients in Uganda were the virus is endemic and more recently appeared in New York City in 1999. A total of 4,200 cases of WNV infections were reported to the Center for Disease Control and Prevention (CDC) in 2002, and by 2003 the number had risen to 9,858 cases including 262 deaths (a 2.66% reported mortality rate). In 2004 and 2005, the reported cases declined (2,282 and 2,949 cases with 77 and 116 fatalities, respectively) [57]. While most (80%) WNV infections occur asymptomatically or with only mild flu-like symptoms without sequelae, in 0.6% of infections, neuro-invasive disease culminating in fatal meningitis or encephalitis can occur, especially in immune-compromised and elderly subjects[58]. In 2002, 23 cases of transfusion- and four cases of organ-transmitted WNV infection were reported and WNV-specific NAT testing was implemented as routine screening in the USA in 2003 [59-62]. Of 27.2 million donations screened, 1,039 viremic donations were identified, with many samples showing low-level viremia [63]. Therefore, WNV NAT is usually performed at the single-donation level in locations and periods with high incidence of infection[64,65]. No TT WNV case has been described so far in Europe, nor did any blood donation test positive for WNV RNA among 62,000 tested samples in the Netherlands[66]. In fact, WNV prevalence is quite infrequent in Europe, probably as a result of viral strain differences, herd immunity, and a relative absence of mosquitoes that can transmit the virus to humans.

Aside from HIV, HCV, HBV and WNV, a number of other viral infections transmitted by transfusion of blood products have been described, even though not all have been associated with clinical manifestation. Human T cell lymphotropic viruses I and II (HTLV-I/II) are associated with adult T cell leukemia and HTLV-associated myelopathy/tropical spastic paraparesis[67]. Both retroviruses have also been attributed a role in the increased risk for developing severe asthma, respiratory and urinary tract infections, uveitis and dermatitis [68-71]. The global epidemiology of HTLV varies widely with prevalence rates up to 10% in certain areas in Japan, and up to 5–6 % in countries of the Caribbean, the Sub-Saharan Africa, and areas in the Middle East and South America[71]. Transfusion-associated transmission of HTLV-I/II occurs through cellular components only and infectivity declines with component storage, particularly after 10 days [72]. The risk of HTLV transmission in the U.S. is very low (1 in 3 million) and cases of HTLV-related disease after transfusion transmission are rare[30].

The transfusion-associated transmission of human herpes viruses, including cytomegalovirus (CMV) and human herpesvirus 8 [HHV-8, also known as Kaposi's Sarcoma-associated herpesvirus] have been described and can pose significant threats, especially to immunocompromised subjects. Like all human herpes viruses, both are cell-associated pathogens and cellular components are thus the main compartment for their transmission by transfused blood products. Recommendations for the control of CMV transmission to susceptible groups have been established and patients at increased risk of CMV disease should only receive CMV-seronegative and/or leucoreduced products, the latter of which has been shown to reduce the risk of CMV transmission considerably [73-76]

HHV8, a human gamma-herpesvirus is the causative agent of Kaposi's Sarcoma, and the probable cause of multicentric Castleman's disease and primary effusion lymphoma[77]. Although normally transmitted through saliva or sexual contact, there is evidence now that HHV-8 can be transmitted via blood transfusion or solid organ transplantation [78-80]. Despite this evidence of successful blood-borne transmission of HHV-8, no data exist yet that link TT HHV-8 transmission to HHV-8-associated diseases in immunocompetent subjects[81,82].

Parvovirus B19 (PV-B19) is a non-enveloped erythrovirus which infects hematopoietic cells. In healthy individuals, PV-B19 infection via the respiratory route leads to erythema infectiosum (Fifth disease), usually a mild and self-limited childhood disease that manifest itself in adults with fever, rash, myalgia, and arthropathy. Pregnant women can transmit the infection intra-uterine with subsequent fetal heart failure and hydrops fetalis. Transfusion transmissions of PV-B19 with mild and non-life-threatening symptoms, even in immuno-compromised patients, have been reported in several studies and epidemiologic analyses have found B19 to be a recurring contaminant of blood products [83-85]. Since the virus lacks an envelope, it is resistant to most virus inactivation methods (solvent/detergent method, heat inactivation or methylene blue)[83,84,86]. In Europe, plasma pools used for production of anti-D immunoglobulin must not exceed 10^4 ^IU of B19 per milliliter and novel, PCR-based detection and quantification kits have been developed to this end in the last few years [87].

Two viruses generally associated with fecal-oral transmission, hepatitis A virus (HAV) and E virus (HEV), have been shown to be at least occasionally transmissible via blood transfusion. Both pathogens are non-enveloped RNA viruses with a low prevalence in developed countries that have advanced environmental hygiene; a vaccine is available for HAV. However, only a few HAV and HEV transfusion-transmissions have been reported, with only mild liver disease as a result [88-91].

There are some other viral agents such as HGV (hepatitis G virus, also known as GB virus type C), TT-virus and SEN virus that have been shown to be transmissible by blood products[92,93]. In a study conducted in Germany in 2004, 1.6% of 25,000 donations screened were found to be HGV RNA positive[94]. Similarly, the prevalence of TTV among healthy blood donors is widespread, especially in Asia (14–36%) [95,96] while the prevalence of SEN-V in healthy individuals ranges from 1.8% (USA) up to 22% in Japan[97]. However, no report exists that links the transfusion-associated transmission of any of these viruses with clinical symptoms and no procedures to protect the blood supply from these pathogens have been implemented.

Aside from the above viral pathogens for which TT related infections have been more or less well documented, there are a number of other potential TT viral threats for which comparable information is missing. Among these, the coronavirus of the severe acute respiratory syndrome (SARS-CoV) or the H5N1 influenza A virus (avian flu) both seem to have primarily respiratory modes of transmission, but evidence of viremia suggests caution and blood-borne transmission has yet to be conclusively ruled out. Especially for SARS-CoV, concerns about potential risk for transfusion transmission led to global implementation of temporary precautionary measures [98]. For relative risk evaluation it will be useful to consider the length of asymptomatic viremic stages in the infected individual, as viruses with only very short viremic episodes along with low levels of viremia may represent relatively moderate risks for TTI, as is the case for WNV.

## Parasites

Parasites are common infectious agents worldwide, and several protozoans have been shown to be transmitted via blood transfusion[99]. Malaria is endemic in tropical and sub-tropical regions of Africa with up to 300 million infections and one million deaths annually[100]. It is caused by one of the four species of *Plasmodium*, (*falciparum*, *vivax*, *malariae *and *oval*) which are mosquito-borne intraerythrocytic parasites that infect liver and red blood cells (RBC) causing periodic episodes of fever and flu-like symptoms, along with massive lysis of erythrocytes. The risk of TT malaria differs widely between low-endemic countries, where the infection is "imported" from outside (e.g. travel to or immigration of individuals from highly endemic regions) and regions of high prevalence of plasmodium infection in the general population. For the latter, only limited data are available, with one report from Benin showing one third of the screened blood donors to harbor *Plasmodium falciparum *trophozoites and potentially be able to transmit the pathogen by blood products[101]. The risk in low-endemic areas is introduced from either travelers to, or immigrants from, high endemic areas[99,102-104]; the latter are usually individuals with a protective immune status who, after many years of infection, can still harbor parasites. Nevertheless, the risk of transfusion-transmitted malaria in low-endemic areas like Europe and the U.S. is low, with only one in 3–4 million units transfused being potentially infectious[105,106]. In some European countries, as well as the U.S., current strategies to prevent TT malaria are based on risk group assessment and include donor deferral for 4–12 months for visitors from low-endemic areas to high-endemic countries, and 3–5 years [or permanently] for donors with a history of residency in an endemic area[105,107,108]. However, this deferral policy leads to an extensive, and for some countries unaffordable, loss of blood donations; this reason is why combined travel-based risk assessment and serological screening tests (enzyme immunoassays, EIA or immunofluorescent antibody test, IFAT) have been introduced in a number of countries [107,109-111].

*Trypanosoma cruzi*, the etiologic agent of Chagas disease (CD), is endemic in Central and South America and parts of Mexico and acute infection can be accompanied with acute symptoms like fever, lymphoadenomegaly or hepato- and spleenomegaly. After 10–40 years, 20–30% of infected patients present with serious organ enlargements (cardiomegaly and occasionally mega-esophagus). Individuals from endemic areas may be chronic carriers of the parasite and are potentially at risk of transmitting the parasite via transfusion of their blood. Although a study performed in the U.S. found a noticeable seroprevalence rate of 0.12–0.20% among such risk donors, blood-borne *T. cruzi *infections are infrequent in North America, with only 7 reported cases. Transfusion cases are well-known in Mexico, Central and South America[112,113]. In many of these countries, serological testing is performed and positive donors are deferred[114]; With the recent licensure of a blood donor screening test, such testing will shortly start in the US.

Other parasites that have been implicated in transfusion-associated transmission are tick-borne pathogens, of which *Babesia microti*, the etiologic agent of babesiosis, is the most frequent transfusion-transmitted parasite. *Babesia microti *is transmitted by *Ixodes *ticks and can lead to severe complications including hemolytic anemia, thrombocytopenia and death, especially when transmitted to an immunocompromised or asplenic subject. There are only few studies regarding transfusion-transmitted babesiosis, including some reporting fatal disease outcome[113], but more than 60 cases are known in the US. Since babesia is an intra-erythrocytic microbe, leucoreduction is an ineffective approach to reduce transmission risk and, given the absence of appropriate serological assays, poses a blood safety risk. *Leishmania donovani*, transmitted primarily by the bite of infected sand flies has also been shown to be transmitted by blood and cause clinical disease in newborns and immunosuppressed subjects. However, these cases appear to be restricted to highly endemic areas such as the Middle East and do not pose significant risks in other parts of the world [115]. Nevertheless, individuals returning to the U.S. from combat zones in Iraq are currently deferred for one year. Aside from parasites, other tick-borne agents such as the two bacteria *Anaplasma phagocytophilum *and *Rickettsia rickettsii *have been rarely found in blood products although most TT tick-borne infections have been largely confined to the Northeastern U.S. and one case of TT babesia infection in Japan [116,117].

## Human prion disease

Variant CJD (vCJD) is the human form of the bovine spongiform encephalitis [BSE]. However, unlike classic Creutzfeldt-Jacob disease (CJD), variant CJD (vCJD) primarily affects people under 50 years of age and is likely to have been transmitted by consuming tissues from BSE-infected animals. Although initially not considered blood-borne, prion diseases have been shown in animal models to be transmissiable by blood products [118]. While evidence for blood-borne transmission of classic CJD is still lacking, three cases of transfusion-transmitted vCJD have been reported in the UK [119-122]. In all three reports, the blood donors developed clinical-apparent vCJD after blood donation. Two of the recipients showed signs of clinical disease whereas the third patient was asymptomatic, but had detectable prions at the time of death (from an unrelated cause). Notably, all three infected patients received non-leucodepleted red blood cells despite the fact that, to date, no TT vCJD case has been associated with the use of plasma products. One important factor to estimate the risk that TT prion diseases may pose in the future is the potentially prolonged pre-clinical carrier state which can last for decades and thus represents a significant risk for transfusion-medicine, at least in regions such as England, Ireland and France where many cases of vCJD have been reported [123]. As a countermeasure, France and the U.K. have adopted the practice to decline individuals who have received a transfusion of any blood component since 1980 indefinitely. Similarly in the U.S. donors with a history of extended residence in the U.K. or Europe, or a history of transfusion in the U.K., are permanently refused.

## Pathogen inactivation

The concept of pathogen inactivation in blood components is to reduce the residual risk of known pathogens and to effectively eliminate new, yet unknown pathogens. However, the different approaches should increase the blood safety without compromising the product efficacy or causing adverse effects, as toxic or mutagenic chemicals may be used in the process. While a number of pathogen reduction methods are employed in Europe, none of them are currently available in the U.S. The choice of a pathogen reduction approach depends on whether it is used to treat components for transfusion such as RBC, PLT and plasma, or for products manufactured from the plasma. In Europe, two distinct methods, methylene blue (MB) and solvent-detergent (SD) are currently employed for the treatment of plasma intended for transfusion. MB is a phenothiazine colorant that inactivates most viruses and bacteria after exposure to visible light. While it has the advantage of being useful for single plasma units, its ineffectiveness against intracellular pathogens and probable interaction with coagulation factors considerably reduce its efficacy[124]. The SD approach acts by disrupting the envelope proteins of targeted pathogens, thus compromising the integrity of the pathogen and rendering it non-infectious. This approach is used on small pools of plasma. The limitation of this technique is that it is not active against non-enveloped pathogens, and that levels of coagulation factors such as protein S may be decreased significantly by some of the SD treatment methods [125,126].

Amotosalen HCL (S-59) is a synthetic psoralen which, when combined with exposure to ultraviolet A [UVA] light, causes a permanent crosslink in bacterial and viral nucleic acid chains, thereby stopping pathogen replication[127]. The photochemical treatment (PCT) with amotosalen and UVA light can be used for FFP and platelets. A commercial product based on this approach is the INTERCEPT system, which has been introduced into clinical practice in Europe a few years ago and has completed phase III trials in the USA [28]. Extensive studies have shown that this approach is effective against all pathogens that are currently screened for, including enveloped and non-enveloped viruses, bacteria (Gram-positive and -negative) and protozoans (*T. cruzi *and *Plasmodium falciparum*) [128-131]. However, a large controlled study has indicated that PCT may have negative effects on the functionality of platelets as transfusions using treated platelet preparations had to be repeated in shorter intervals than when using untreated platelet preparations [132]. Aside from these approaches to pathogen reduction in platelet preparations, at least three techniques for pathogen inactivation in RBC are currently under development: S-303, a synthetic alkylating agent, (a compound of the frangible anchor-linked effectors [FRALE] class) capable of disrupting pathogen RNA or DNA[133]; Inactine, a binary ethyleneimine that binds to nucleic acids resulting in the inhibition of pathogen replication [134-137]; and riboflavin [vitamin B_2_], a naturally occurring nutrient [138-143]. Even though some of these agents have entered phase III clinical trials none of them have been officially approved at the time this review was written. These approaches, as well as newly developed ones, will need to show convincingly that they successfully eliminate targeted pathogens while maintaining blood product quality. In addition, possible limitations, such as high costs, long-term side effects of some additives and inability to inactivate certain pathogens like spore-forming bacteria will need to be overcome to ensure pathogen-free blood products on a large scale and at affordable prices.

## Conclusion

The general public may be idealistic in their belief that risk-free blood products are achievable in today's world. In fact, the threat of infectious agents entering the blood supply is not static and may evolve as new pathogens emerge or as old ones change their epidemiological pattern. Nevertheless, the goal of a safe and affordable blood supply that can meet the growing global demands may be reached by the coordinated optimization of each step in the transfusion chain, including the careful consideration of donor eligibility criteria, adherence to rigorous rules during donation, processing and storage, the optimal implementation of available screening tests, the use of suitable pathogen inactivation methods and finally the vigilance of prudent physicians, who evaluate the necessity of each transfusion. Efforts invested in providing lowest possible risk blood products need to be matched by the diligence of physicians administering the transfusions who need to report adverse consequences of blood transfusions. Hence, national haemovigilance systems linked to an international network are becoming indispensable elements of blood product safety and quality. Combined with the development and implementation of sensitive and affordable detection and inactivation approaches, these measures can make blood transfusion a safer form of therapy even in places where the risks to date have to be considered significant [5,6,144,145].

## Conflict of interest

The author(s) declare that they have no competing interests.
